# Estimating the Population Size of Female Sex Workers in Three South African Cities: Results and Recommendations From the 2013-2014 South Africa Health Monitoring Survey and Stakeholder Consensus

**DOI:** 10.2196/10188

**Published:** 2018-08-07

**Authors:** Michael A Grasso, Albert E Manyuchi, Maria Sibanyoni, Alex Marr, Tom Osmand, Zachary Isdahl, Helen Struthers, James A McIntyre, Francois Venter, Helen V Rees, Tim Lane

**Affiliations:** ^1^ Institute for Global Health Sciences University of California, San Francisco San Francisco, CA United States; ^2^ Anova Health Institute Johannesburg South Africa; ^3^ Division of Infectious Diseases & HIV Medicine Department of Medicine University of Cape Town Cape Town South Africa; ^4^ School of Public Health & Family Medicine University of Cape Town Cape Town South Africa; ^5^ Wits Reproductive Health and HIV Institute University of Witwatersrand Johannesburg South Africa

**Keywords:** female sex workers, population size estimation, integrated biological and behavioral surveillance surveys, South Africa, HIV

## Abstract

**Background:**

Robust population size estimates of female sex workers and other key populations in South Africa face multiple methodological limitations, including inconsistencies in surveillance and programmatic indicators. This has, consequently, challenged the appropriate allocation of resources and benchmark-setting necessary to an effective HIV response. A 2013-2014 integrated biological and behavioral surveillance (IBBS) survey from South Africa showed alarmingly high HIV prevalence among female sex workers in South Africa’s three largest cities of Johannesburg (71.8%), Cape Town (39.7%), and eThekwini (53.5%). The survey also included several multiplier-based population size estimation methods.

**Objective:**

The objective of our study was to present the selected population size estimation methods used in an IBBS survey and the subsequent participatory process used to estimate the number of female sex workers in three South African cities.

**Methods:**

In 2013-2014, we used respondent-driven sampling to recruit independent samples of female sex workers for IBBS surveys in Johannesburg, Cape Town, and eThekwini. We embedded multiple multiplier-based population size estimation methods into the survey, from which investigators calculated weighted estimates and ranges of population size estimates for each city’s female sex worker population. Following data analysis, investigators consulted civil society stakeholders to present survey results and size estimates and facilitated stakeholder vetting of individual estimates to arrive at consensus point estimates with upper and lower plausibility bounds.

**Results:**

In total, 764, 650, and 766 female sex workers participated in the survey in Johannesburg, Cape Town, and eThekwini, respectively. For size estimation, investigators calculated preliminary point estimates as the median of the multiple estimation methods embedded in the IBBS survey and presented these to a civil society-convened stakeholder group. Stakeholders vetted all estimates in light of other data points, including programmatic experience, ensuring inclusion only of plausible point estimates in median calculation. After vetting, stakeholders adopted three consensus point estimates with plausible ranges: Johannesburg 7697 (5000-10,895); Cape Town 6500 (4579-9000); eThekwini 9323 (4000-10,000).

**Conclusions:**

Using several population size estimates methods embedded in an IBBS survey and a participatory stakeholder consensus process, the South Africa Health Monitoring Survey produced female sex worker size estimates representing approximately 0.48%, 0.49%, and 0.77% of the adult female population in Johannesburg, Cape Town, and eThekwini, respectively. In data-sparse environments, stakeholder engagement and consensus is critical to vetting of multiple empirically based size estimates procedures to ensure adoption and utilization of data-informed size estimates for coordinated national and subnational benchmarking. It also has the potential to increase coherence in national and key population-specific HIV responses and to decrease the likelihood of duplicative and wasteful resource allocation. We recommend building cooperative and productive academic-civil society partnerships around estimates and other strategic information dissemination and sharing to facilitate the incorporation of additional data as it becomes available, as these additional data points may minimize the impact of the known and unknown biases inherent in any single, investigator-calculated method.

## Introduction

Female sex workers (FSWs) have long been recognized as a key population at a high risk for HIV infection [1,2]. In the context of a generalized HIV epidemic in South Africa, individual and structural factors such as poverty, stigma, discrimination, and criminalization of sex work contribute to FSWs’ vulnerability to HIV and complicate efforts to control the HIV epidemic in the sex worker population [3]. Although South Africa still criminalizes sex work, FSW populations are a visible, mobilized, and economically significant population across the country, including the major metropolitan areas that are centers of industrial and trade-based employment, provincial cities and towns, and rural areas, particularly those traversed by the country’s well-developed national highway network that links Atlantic and Indian Ocean port cities to the South African interior as well as the landlocked countries to South Africa’s north [4]. FSWs work in diverse settings, including along major transport routes, at public venues such as urban street corners, parks, bars, and taverns, as well as in more closed spaces such as private homes, where they mainly interact with clients using social media platforms [4].

HIV surveillance data, including population size estimates (PSEs) on the South African FSW population, are limited. Studies conducted in the 1990s and 2000s observed that as many as half of all sampled sex workers were HIV positive, but these studies did not include PSEs [5,6]; recent South African initiatives aimed to meet the HIV needs of key populations, including those sponsored by the US President’s Emergency Plan for AIDS Relief (PEPFAR) and the Global Fund to Fight AIDS, Tuberculosis and Malaria (the Global Fund), have highlighted the need for reliable, methodologically rigorous PSEs for key populations generally and FSWs in particular. In 2013, fieldwork undertaken by the South African National AIDS Council (SANAC) and sponsored by the Global Fund estimated that there were roughly 150,000 FSWs in South Africa or nearly 1% of the adult female population aged 15-49 years [7]. Despite the explicit inclusion of FSWs in South Africa’s national HIV strategic plans since at least 2007, prior to 2016, these efforts had not been informed by rigorously collected surveillance or survey data to quantify HIV treatment or biomedical prevention for the FSW population.

In 2013-2014, in partnership with South Africa’s National Department of Health (NDOH) and SANAC, PEPFAR and the US Centers for Disease Control and Prevention (CDC) sponsored a collaboration between the University of California San Francisco, Anova Health Institute, and the Wits Reproductive Health and HIV Institute to conduct the South Africa Health Monitoring Survey (SAHMS), an integrated biological and behavioral surveillance (IBBS) survey, in South Africa’s three largest cities of Johannesburg, Cape Town, and eThekwini. The SAHMS aimed to estimate HIV prevalence and associated risk, prevention, and health-seeking behaviors among FSWs as well as to estimate the size of the FSW population in each of the three metropolitan areas. HIV prevalence and behavioral results have been reported elsewhere [2]. Briefly, we estimated that 71.8% (95% CI 56.5-81.2) of FSWs in Johannesburg, 39.7% (95% CI 30.1-49.8) in Cape Town, and 53.5% (95% CI 37.5-65.6) in eThekwini were HIV infected. Among HIV-positive FSWs, only 26.9% in Johannesburg, 23.6% in Cape Town, and 35.3% in eThekwini were on antiretroviral treatment.

As there is no “gold standard” for estimating the size of key populations, we adapted CDC-recommended best practices [8] by integrating multiple multiplier-based methods of estimating the size of the FSW population at each site into the IBBS surveys and by engaging in a participatory process to achieve stakeholder consensus PSEs. In this paper, we have described these survey methods, PSE methods and results, and the consensus process through which FSW stakeholders adopted PSEs and plausible ranges (PRs) for purposes of strategic planning, policy making, advocacy, and programming.

## Methods

### Sample Size and Precision

The SAHMS was a cross-sectional HIV bio-behavioral surveillance study with a target sample size of 500 FSWs in each city. We used respondent-driven sampling (RDS) methods [9-12] that have been subsequently adapted for key populations HIV surveillance and population size estimation purposes [13-19]. We have described elsewhere how RDS recruitment operated in the SAHMS [2]. Briefly, each city’s sample was recruited independently of the others’. Recruitment of each sample began with 1-3 seeds identified by stakeholders and study staff during pre-IBBS formative assessment; each seed recruited up to 3 additional FSWs from their social and professional networks, who recruited up to 3 additional FSWs, and so on in Markov chains, as shown in Table 1.

The study procedures consisted of a behavioral survey and biological testing for HIV. All participants who wanted to know their HIV status were offered rapid HIV testing services (HTS). Eligible candidates were those who were born biologically female; aged 16 years or older; had exchanged sex for money with someone other than a primary partner in the previous 30 days; and had lived, worked, or socialized in the urban area where they were recruited for the previous 6 months. Participants provided written informed consent for study procedures and separate written informed consent for rapid HTS (per South African guidelines). HIV-positive FSWs were referred to FSW-competent, nonstigmatizing clinical care. Survey data collection commenced in July 2013 and concluded in February 2014.

Laboratory and statistical analyses of biological and behavioral survey data followed the Strengthening the Reporting of Observational Studies in Epidemiology RDS guidelines [20], and the full description of laboratory methods has been provided in the SAHMS final report [2]. In the next sections, we have described the background and methodological approach to each population size estimation method.

#### Wisdom of the Crowds

The theoretical assumption of “wisdom of the crowds” (WOTC) asserts that a reasonable estimate of the size of a population may be derived from aggregating responses from survey participants [21]. The SAHMS included the following question: “Approximately how many other women who have sex for money do you think live in and around [survey city]?” To improve response reliability, the question was asked twice within the survey. The final estimate was reached by taking the average of the two median estimates and ranges.

#### Unique Object Multiplier

The unique object multiplier is a 2-step method commonly used in conducting population size estimation of key populations. The first step involves distributing unique, memorable objects in advance of the survey throughout the study area to the members of the population of interest. The objects were determined through stakeholder consultation in each city. In eThekwini, lavender-colored bracelets were distributed, while compact make-up kits were used in Johannesburg and Cape Town. In each city, study staff and stakeholder volunteers distributed objects to FSWs throughout the study area a few weeks prior to survey launch, varying days and times in order to achieve the largest distribution.

To avoid distribution biases and errors in the first step of this process, we relied on the advice of individual volunteers and staff who were familiar with the local FSWs, or who were themselves local FSWs, to minimize the possibility that individuals would receive multiple objects or that objects would be distributed to nonpopulation members. The numbers of objects distributed at a particular time and geographic area (eg, street intersection, brothel) were recorded and varied to ensure that different individuals and subpopulations would be encountered in each object distribution event. Finally, with each brief interaction, staff screened women to verify their FSW status and whether they had previously received the object.

The second step was an item in the survey instrument: “In the previous 6 months, did you receive an object, like the one I am showing you now?” with the interviewer holding up an example of the object distributed. The proportion of survey respondents who answered “yes” to the question was used to calculate the RDS-adjusted size estimate for this method. The calculation used for this method was N=n/p; where “N” is the PSE, “n” the number of objects distributed in the population, and “p” the proportion of participants who reported receiving an object in the survey.

#### Unique Event Multiplier

The 2-step principles and calculation for the unique event multiplier are similar to the unique object. In the first step, in advance of the survey launch in each city, staff and stakeholders sponsored a memorable launch event, with the theme and name of the event determined through stakeholder input in each city and the event publicized through FSW stakeholders and social networks. Staff and stakeholders counted each woman who entered the event and screened all women to confirm FSW status. Each count was recorded; discrepancies between counters were resolved through discussion until a count deemed to be reasonable was arrived at by all counters. In the second step, survey participants were asked if they attended the event, with the event identified by its name and date. To calculate an RDS-adjusted PSE, we used the previously mentioned formula N=n/p: here “n” is the number in attendance at the event and “p” the proportion of the survey sample who reported having attended the event.

#### Service Multiplier

In this 2-step process, staff first obtained de-duplicated counts of FSWs who utilized any clinical HIV or community-based service (eg, HIV testing, attendance at an advocacy workshop) from partnering stakeholder organizations between January 1 and June 16, 2013. In Johannesburg, these were visits to Esselen Street Clinic, a clinic operated by clinical staff at the Wits Reproductive Health and HIV Institute, where the visiting population primarily comprises sex workers; in Cape Town and eThekwini, these were either having attended a “Creative Space” advocacy workshop organized by the Sex Worker Education and Advocacy Taskforce or having received HTS through the TB/HIV Care Association, who provide mobile testing to FSWs. In the second step, the survey asked participants whether they had received the particular service between January 1 and June 16 (with January 1 referenced as “New Year’s Day” and June 16 as “Youth Day,” a South African public holiday and, therefore, a salient recall endpoint). With the same N=n/p multiplier formula; here “n” is the number of de-duplicated FSWs reported by the service provider and “p” is the proportion of participants who reported receiving services from the given provider.

**Table 1:** Respondent-driven sampling sample size and recruitment statistics for three samples of female sex workers in South Africa.

Site	Sample size	Seeds	Waves to equilibrium	Total waves	Mean network size
Johannesburg	764	5	7	17	20.67
Cape Town	650	6	10	29	16.98
eThekwini	766	3	6	16	11.40

#### Calculation of Preliminary Population Size Point Estimates

Study investigators calculated a point estimate for the FSW population in each city that was the median of a plausible range of individual point estimates derived from the sources described above. Investigators excluded point estimates as implausible in calculating the median if they were outside of an obvious range of reasonableness—for example, a preliminary point estimate could not be less than the survey sample size in each city, or it would suggest that more than half the adult female population were engaged in sex work. The investigators adopted the median of the plausible estimates as the preliminary PSE, with the largest reasonable point estimate as an upper plausibility bound and the lowest reasonable point estimate as the lower plausibility bound.

#### Modified Delphi Process and Adoption of Consensus Population Size Estimates

Using this range of estimates, investigators then invited input on the preliminary PSEs, including their *a priori* exclusion of implausible results, from a stakeholder committee following a consensus process described by colleagues in the San Francisco Department of Public Health [22] and previously implemented in Tanzania [23] and Ghana [24]. The study investigators convened a meeting with stakeholders who were familiar with the three FSW populations to present the preliminary PSEs and associated upper and lower plausible bounds. The stakeholder group included representatives of NDOH, civil society human rights advocacy and health services organizations represented on the SANAC, and other academic experts. The PSE and crude data were distributed to stakeholders in advance of an in-person stakeholder meeting.

At this meeting, investigators reviewed all the individual PSE methods outlined above, discussed the variation between and limitations of each method, and identified their *a priori* implausible estimates. Upon achieving consensus on the plausible range of PSEs, the investigators calculated preliminary median PSEs and upper and lower plausible bounds. Preliminary PSEs were also compared with census data from 2011 to back-calculate the proportion of the adult female population engaging in sex work in each city to demonstrate where the estimate lay within a range of reasonableness, including comparison to other PSE studies and assumptions from other contexts. In this case, the group considered PSEs derived from a 2013 national rapid assessment of the sex worker population commissioned by SANAC and presented by Konstant et al [7] to assess whether the preliminary median PSEs and PRs were sensitive to the previous results. (Briefly, the rapid assessment’s multimethod approach consisted of mapping and enumeration, interviews with sex workers, focus group consultations with key informants, and fieldwork counts conducted by stakeholder fieldworkers. Results were reported as counts and proportions of the adult female population aged above 15 years.

Finally, the investigators facilitated a stakeholder group discussion to compare the preliminary median PSEs and plausibility ranges against stakeholders’ own experiences of engagement with the FSW population through existing prevention or treatment programs. This process provided the opportunity to reconsider any point estimates that investigators had excluded *a priori*. At the conclusion of the meeting, the group was invited to reject, amend and recalculate, or adopt the preliminary PSEs as consensus PSEs.

### Data Analysis

We calculated HIV prevalence and other uni- and bivariable proportions using the RDS Analysis Tool version 7.1.46 and the SPSS version 23.0. Each sample’s results were analyzed, weighted, and reported independently of the others. We estimated the size of the FSW population in each city following best practices that recommend multiple methods and “multiple multipliers” [8] and following a 2-phase data triangulation and consensus-based process.

## Results

### Sampling or Recruitment

We recruited 2180 FSWs across the three sites. In Johannesburg, recruitment began in August 2013 and continued for 25 weeks, recruiting a total of 764 women through 5 seeds. The Cape Town site launched in July 2013 and was open for 28 weeks, with a final sample of 650 through 6 seeds. The eThekwini study site began recruiting participants in September 2013 and was operational for 22 weeks, with 766 women included in the final sample recruited through 3 seeds.

PSEs for each city and the survey counts on which they are based, for example, the count of participants in the survey who recalled receiving the unique object, have been listed by estimation method in Table 2. In Johannesburg, the WOTC produced the lowest estimate at 3000 FSWs (range 3000-3500) and was ultimately deemed implausibly low by consensus and excluded from calculation of the median. The unique object had the highest estimate at 10,895 FSWs (95% CI 582-25,018). The unique event produced an estimate of 4500 FSWs (95% CI 272-not applicable). The service multiplier result was deemed an unreasonably low estimate as it produced an estimate equal to the survey sample size. Previously published literature has estimated the Johannesburg FSW population at 10,894 [7].

In Cape Town also, WOTC produced the lowest point estimate at 1500 FSWs (range 1000-1750) and unique object the highest at 23,750 FSWs (95% CI 783-59,375). This value was deemed outside the range of plausibility by stakeholder consensus and was excluded from calculation of the median. The unique event multiplier result was 7500 FSWs (95% CI 1380-37,500). The two service multiplier results in Cape Town were 4579 FSWs (95% CI 3153-6869) and 2551 FSWs (95% CI 1708-3585). Previously published literature has estimated the Cape Town FSW population at 7351 [7].

In eThekwini, the WOTC estimate was 4000 FSWs (range 3000-5000). The unique object multiplier result was 11,200 FSWs (95% CI 326-34,000). The unique event resulted in an estimate of 747 FSWs. However, this estimate was judged to be highly implausible since it was well below the de-duplicated data provided by service providers and, therefore, excluded from the final analysis. This is very likely attributable to a misunderstanding regarding the unique event attendance question among eThekwini survey participants. The two service multiplier estimates were 12,840 FSWs (95% CI 7379-33,879) and 9323 FSWs (95% CI 5255-17,515). Prior literature has estimated the FSW population in this city at 6145 [7].

The Modified Delphi consensus process meeting with stakeholders endorsed the investigator recommendations on preliminary point estimates (median of all estimates), resulting in the exclusion of unreasonable results from calculating the median. In Cape Town, WOTC was dismissed as implausible based on program data and expert opinion. The point estimate became the median of the remaining estimates, rounded up. Stakeholders were given the option of accepting the highest and lowest plausible estimate as the PR; in Cape Town and eThekwini, they relied on expert opinion to round the upper boundary down.

### Population Size

Table 2 presents preliminary and consensus PSEs and PR results, including the proportion of the adult female population represented by the consensus PSEs and PRs. We have included Konstant et al’s results to demonstrate the sensitivity of the IBBS-derived consensus PSEs to previous estimates [7].

**Table 2:** Consensus population size estimates of South African female sex workers (FSWs) in the South Africa Health Monitoring Study 2013-2014.

City and method		FSW count, n	Sample proportion, p	Point estimate, N (95% CI or range)	Final estimate, n (%)^a^	Plausible results, range (%)
**Johannesburg**						
	Wisdom of the crowds	N/A^b^	N/A	3000^c^	7697 (0.48)	5000-10,895 (0.31-0.69)
	Unique object	1351	0.124	10,895 (582-25,018)		
	Unique event	27	0.006	4500 (272-N/A)		
	Service multiplier	261	0.341	765^c^		
	Literature	N/A	N/A	10,894		
**Cape Town**						
	Wisdom of the crowds	N/A	N/A	1500^c^	6500 (0.49)	4579-9000 (0.35-0.69)
	Unique object	950	0.04	23,750^c^		
	Unique event	75	0.01	7500 (1380-37,500)		
	Service multiplier 1	577	0.126	4579 (3153-6869)		
	Service multiplier 2	398	0.156	2551 (1708-3585)		
	Literature	N/A	N/A	7351		
**eThekwini**						
	Wisdom of the crowds	N/A	N/A	4000 (3000-5000)	9323 (0.77)	4000-10,000 (0.33-0.83)
	Unique object	952	0.075	11,200 (326-34,000)		
	Unique event	56	0.085	747^c^		
	Service multiplier 1	642	0.05	12,840 (7379-33,879)		
	Service multiplier 2	578	0.062	9323 (5255-17,515)		
	Literature	N/A	N/A	6145		

^a^% adult female population.

^b^N/A: not applicable.

^c^Implausible estimate not used in the calculation of median preliminary population size estimate.

## Discussion

### Principal Results

The SAHMS study, and the PSEs derived from it, fill a critical strategic information gap by providing conservative yet robust PSEs of FSWs in South Africa’s three largest cities of Johannesburg, Cape Town, and eThekwini, producing point estimates of 7697, 6500, and 9323, respectively.

### Strengths

This study is, to our knowledge, the first published study of its kind for South Africa where the incorporation of stakeholder consensus into the analysis of IBBS data was an integral component of the population size estimation methodology. Indeed, the service multiplier methods could not be implemented without significant stakeholder engagement, and stakeholder endorsement of the PSE results as plausible is critical to the PSEs’ utility. In this case, stakeholder endorsement of these PSEs was critical to NDOH and SANAC developing, launching, and costing the National Sex Worker HIV Plan 2016-2019 [25] as well as setting realistic and data-informed FSW prevention and treatment targets for South Africa’s HIV/STI National Strategic Plan 2017-2022 [26]. While these planning processes were entirely independent of SAHMS data collection or its PSE processes, stakeholders’ decision that surveillance data and PSEs were reliable enough to inform strategic planning was only possible because they were meaningfully and consistently engaged with the data collection and interpretation process.

### Comparison With Prior Work

The estimates derived from our methodology in these cities are largely consistent with 2013 estimates by Konstant et al, derived from different methodologies [7]. While stakeholders acknowledged that the PSEs appeared to be lower than they had expected (a result also reported by Konstant et al), stakeholders were persuaded to rely on these results as they were based upon empirical methodologies that were consistently and transparently applied to the IBBS PSE data. Thus, these consensus PSEs were acknowledged by stakeholders to be data informed and usable for their purposes of programmatic planning and benchmarking.

### Limitations

We are aware that the major critique and limitation of the individual methods we used, as well as the consensus process through which final PSEs were calculated and adopted, are that the methods and process are subject to significant and frequently unmeasurable biases, making it difficult to impossible to assess PSE accuracy and subjects’ precision to subjective biases. In fact, we substantially agree and would contend that while greater accuracy is of course a goal, it is unlikely to be achieved through a single method with enough rigor to achieve scientific consensus on bias and accuracy anytime soon. The virtue of the individual PSE methods and the consensus process described in this paper lies in their utility to public health planning and action. Individually, the multiplier methods that we selected for inclusion in the SAHMS are available, easy to implement, rigorous enough to be reproducible, and—critically—transparent in their limitations and are generally easily understood by stakeholders. Moreover, numbers that do not align with stakeholder opinion or experience are not likely to be adopted or utilized, which essentially throws good money after bad. None of this should be interpreted as our endorsement of methodological sloppiness or indiscriminate guessing; it is simply a recognition that lives are at stake and avoidable infection, illness, and death should be prioritized over methodological debates in the meantime.

These FSW PSEs are also subject to several methodological and implementation-related limitations. As discussed previously, reasonable people may disagree on whether the results are accurate or precise *enough*, and we acknowledge that there is no empirical way to validate consensus point PSEs. Nearly every step in the process is vulnerable to biases introduced through both random and human error; as facilitators of the consensus process, investigators have a duty to be ruthlessly and transparently skeptical of *all* results in light of other available evidence and stakeholder experience so that reversion to the mean of empirically collected and analyzed data is privileged over indiscriminate guessing. In particular, we are aware of the emerging consensus in the scientific community that Delphi methods such as WOTC have become less necessary or desirable to be included in multimethods comparisons. We report it here only because it was a method considered by this stakeholder group in 2016, and the purpose of this paper is to describe stakeholder consensus methodology and the results generated through it, more than to validate or invalidate any individual PSE methodology. We are aware of the major empirical limitations of similar Delphi methods; they have been perhaps less robust than, for example, multiplier methods. We substantially agree, and there may be enough, more empirical and robust, methodologies now available that a recommendation to exclude them in the future would not be unwarranted. This said, we note that as implemented and analyzed in SAHMS, WOTC produced the lowest point PSEs compared with the capture-recapture multiplier methods, considered more empirically based.

These consensus PSEs are primarily informed by point estimates from the more empirically satisfying and theoretically reproducible multiplier methods, yet we caution that even these point estimates must be understood and qualified as being subject to several biases embedded in these methods. For example, it is not possible to independently validate that unique object or event counts include only individuals who are true population members. Additionally, given the requirement that multiplier counts be independent of survey counts, even the most rigorous implementation of multiplier and survey methods cannot guarantee plausible results as demonstrated by Cape Town’s object multiplier. Self-report bias may have been introduced in multiplier methods relying on socially desirable affirmative answers to questions about, for example, being in possession of a make-up kit (object) or getting HIV tested in the last 6 months (service). Additionally we observed relatively low attendance at each of the three unique events, and in the case of eThekwini, the number of attendees recaptured through RDS recruitment produced an implausible result nearly equal to the site’s achieved sample size (ie, ~100% recapture). For all these reasons, it is advisable to discuss proposed multiplier method procedures with the population during presurvey assessments such as phrasing of recapture survey questions to avoid misunderstandings and biased responses. Furthermore, it is important to monitor and document the implementation of both sides of the capture-recapture methods carefully. In the absence of these recommendations, it may otherwise not be possible for investigators or stakeholders to make reasoned, qualitative judgments about the plausibility of the individual results or the range of preliminary PSE results.

Additionally, it is debatable as to whether venue-based nonprobability and quasi-probability methods may provide more reliable population size data for purposes of estimating unmet HIV program needs; in particular, Rao et al’s [27] side-by-side comparison of the advantages and limitations of RDS with venue-based nonprobability sampling provides critical perspective on clearly defining a target population, if assessing unmet service delivery needs for service delivery is among the intended outcomes or uses of PSE data. We acknowledge the potential advantages of such methods particularly in resource-limited settings, especially because strategic information-gathering resources are finite and increasingly constrained, but we believe that currently, even in a human rights-protecting legal environment such as South Africa’s, stigma and discrimination, as well as sex workers’ well-founded fears of legal jeopardy and human rights violations by law enforcement (sex work itself remains criminalized), may prevent some FSWs (and other key populations members) with substantial unmet needs from being visible at selected, relatively public hotspots where they might be systematically enumerated. Similarly, nonservice delivery venues where FSWs are likely to be enumerated (eg, brothels, the internet) may be more difficult for investigators to access than for RDS recruitment to penetrate. The chief advantage of RDS with key populations—that it relies on network ties within a population to populate the sample—requires that it be implemented with substantial baseline knowledge of the population’s characteristics and needs. Here stakeholder perspectives are critical to informing investigators’ perspectives, and population members may also properly be considered stakeholders in a consensus process, even if they are not sitting in a conference room with service provider and other types of stakeholders, whose perspectives may inherently be biased toward those who are countable and have already been reached. In this sense, failure to demonstrate substantial network transition out of service provider-related networks suggests either optimal service coverage of the population (highly improbable in sex work-criminalized environments) or methods-implementation limitations that must be identified and acknowledged in analysis.

Successive sampling (SS)-PSEs are possible to calculate from RDS data [28] and, on their face, may appear more methodologically and empirically satisfying. We did not include SS-PSEs here only because these have not been vetted by this stakeholder group, and the participatory stakeholder process is the subject of this paper as much as the estimates it produced. We endorse SS-PSE’s inclusion in multiple-method comparisons of future surveillance and population size estimation work in South Africa and elsewhere. SAHMS II, which will be fielded in 2018-19, will calculate SS point estimates and present these for consideration by stakeholders for calculating a mean PSE and reaching consensus PSEs. SS-PSE accuracy and precision are dependent on well-monitored field implementation of RDS and proper post-hoc accounting of bias in RDS recruitment data. For this reason, we could not recommend reliance on any single method and continue to endorse vetting and triangulation of multiple empirical methodologies by stakeholders and technical experts in a participatory process.

### Lessons Learned

At the end of the day, a PSE has no inherent value unless it is adopted and used consistently by all stakeholders in government, civil society, and Global Health financing partners. Investigators cannot hope to achieve anything like accuracy without the granular knowledge that local stakeholders possess regarding FSWs and similarly stigmatized and hidden key populations; stakeholders cannot make this judgment of a PSE result unless they judge the method of producing it to be reasonable, transparent, and competently applied. Ultimately, our method places great responsibility in the hands of technical advisors who must navigate advocacy, service provider, and political interests while privileging empirically derived data in weighing what is and is not a reasonable result, even when this is inconvenient. The authors hope to have ably discharged this duty both in reporting these first consensus-based PSEs for South African FSWs and in describing the process through which the consensus was achieved. Because the identification of a “gold standard” methodology that can consistently produce a single, accurate result for key populations like FSWs continues to elude us all, we recommend this approach that incorporates multiple empirical methods into a “multiple multipliers” comparison and facilitates participatory data triangulation to achieve stakeholder consensus PSEs. Presently, HIV strategic planning efforts in South Africa and throughout the world involve costing of the proven but expensive biomedical prevention and treatment technologies that are essential to achieving real and lasting impact on the high-prevalence, high-incidence epidemics experienced by FSWs and other key populations. The experience of South Africa suggests that these consensus PSEs have provided a necessary and useful baseline from which to launch an evidence-informed assault to end key populations’ HIV epidemics.

## Abbreviations

CDC: US Centers for Disease Control and Prevention
FSW: female sex worker
HTS: HIV testing services
IBBS: integrated biological and behavioral surveillance
NDOH: National Department of Health
PEPFAR: President’s Emergency Plan for AIDS Relief
PR: plausible range
PSE: population size estimate
RDS: respondent-driven sampling
SAHMS: South Africa Health Monitoring Survey
SANAC: South African National AIDS Council
SS: successive sampling
WOTC: wisdom of the crowds
